# Optimization of Magnetoplasmonic *ε*-Near-Zero Nanostructures Using a Genetic Algorithm

**DOI:** 10.3390/s22155789

**Published:** 2022-08-03

**Authors:** Felipe A. P. de Figueiredo, Edwin Moncada-Villa, Jorge Ricardo Mejía-Salazar

**Affiliations:** 1 Instituto Nacional de Telecomunicações (Inatel), Santa Rita do Sapucaí 37540-000, Brazil; felipe.figueiredo@inatel.br; 2 Escuela de Física, Universidad Pedagógica y Tecnológica de Colombia, Avenida Central del Norte 39-115, Tunja 150003, Colombia; edwin.moncada@uptc.edu.co

**Keywords:** genetic algorithm optimization, magnetoplasmonics, magneto-optics, sensing, TMOKE

## Abstract

Magnetoplasmonic permittivity-near-zero (ε-near-zero) nanostructures hold promise for novel highly integrated (bio)sensing devices. These platforms merge the high-resolution sensing from the magnetoplasmonic approach with the ε-near-zero-based light-to-plasmon coupling (instead of conventional gratings or bulky prism couplers), providing a way for sensing devices with higher miniaturization levels. However, the applications are mostly hindered by tedious and time-consuming numerical analyses, due to the lack of an analytical relation for the phase-matching condition. There is, therefore, a need to develop mechanisms that enable the exploitation of magnetoplasmonic ε-near-zero nanostructures’ capabilities. In this work, we developed a genetic algorithm (GA) for the rapid design (in a few minutes) of magnetoplasmonic nanostructures with optimized TMOKE (transverse magneto-optical Kerr effect) signals and magnetoplasmonic sensing. Importantly, to illustrate the power and simplicity of our approach, we designed a magnetoplasmonic ε-near-zero sensing platform with a sensitivity higher than $56^{\circ }/\mathrm{RIU}$ and a figure of merit in the order of $10^{2}$. These last results, higher than any previous magnetoplasmonic ε-near-zero sensing approach, were obtained by the GA intelligent program in times ranging from 2 to 5 min (using a simple inexpensive dual-core CPU computer).

## 1. Introduction

Plasmonic nanostructures are at the core of recent transformative breakthroughs in a diverse set of areas such as biosensing [1], waveguiding [2], and energy harvesting [3]. These nanostructures derive their unique properties from surface plasmon resonances (SPRs), i.e., the resonant coupling of optical fields with surface charge density oscillations (on metal surfaces), allowing light to be trapped, routed, and manipulated at nanometer-length scales [4,5]. It is precisely these latter features that have motivated an increasing interest in the use of plasmonics despite the intrinsic resistive losses of metals. Recent achievements include highly integrated plasmonic waveguides [6], nanorouters [7], demultiplexers [8], and nanoplatforms for single-molecule studies [9]. In contrast to plasmonic nanoparticles and nanogratings, where light can be directly coupled with nanoparticle resonances (called localized surface plasmon resonances) [10] or through diffraction modes [11], respectively, bulky prism couplers (for attenuated total reflection mechanism) are required for flat metal surfaces. Attempts to overcome this last drawback include the use of thin or ultrathin films of permittivity (ε) near-zero (ε-near-zero) materials [12], promising high miniaturization levels. However, the broad resonance peaks of this approach inhibit sensing applications due to the high overlap of nearby resonances, limiting applications mainly to broadband electromagnetic absorbers [13,14,15].

On the other hand, magneto-optical effects in magnetophotonic nanostructures, i.e., nanostructures containing materials with magneto-optic (MO) activity [16], have been widely used to improve the resolution capabilities of plasmonic biosensing platforms [17,18,19,20,21]. In particular, the transverse magneto-optical Kerr effect (TMOKE), defined as the relative change in the reflected light amplitude $R_{p}$ (the subindex *p* indicates that this MO effect only exists for *p*-polarized light) when the magnetization of the system (**M**) is flipped

(1) $$\mathrm{TMOKE}=\frac{R_{p}\left(+M\right)-R_{p}\left(-M\right)}{R_{p}\left(+M\right)+R_{p}\left(-M\right)}$$

exhibit sharp Fano-like peaks around the plasmonic resonance angle/wavelength. These sharp resonances are due to small magnetically tuned shifts of the resonance conditions, which are used instead of wide plasmonic peaks to enable high-resolution detection of minute refractive index changes (associated to adsorption processes) at the sensing surface [20]. Indeed, merging ε-near-zero with TMOKE can be used for new highly integrated biosensing platforms, as has been recently shown [22,23,24].

Despite the advantages of MO-ε-near-zero biosensing platforms, their detection capabilities have not yet been developed. The sensitivity and TMOKE amplitudes of these nanostructures largely depend on the structural design and the angle of incidence (θ) to meet the phase-matching condition [22]. Contrary to conventional plasmonic surfaces, where an analytical expression for the phase matching condition is available, the tuning of MO-ε-near-zero resonant layer thickness (*d*) and θ is performed through the tedious and time-consuming manual processing of large sets of data [23,24]. In addition, manual tuning methods do not guarantee jointly optimized values for *d*, θ, and TMOKE, which, in turn, affect the corresponding performance of the structure. In this work, we developed a genetic algorithm (GA) combined with the scattering matrix method (SMM) for the design of optimized ε-near-zero-based magnetophotonic nanostructures. GAs are stochastic search optimizers that offer a flexible and simple yet powerful way for parameter optimization based on evolutionary and natural selection principles. In particular, GAs operate exceptionally well in dealing with multiparametric optimization problems [25,26,27,28]. Hence, we exploited this last feature in two different ways. First, we adopted the TMOKE value as the fitness function to search for the optimum thickness, *d*, and angle of incidence, θ, that produced $\left|\mathrm{TMOKE}\right|\approx 1$, demonstrating that magnetoplasmonic devices with extraordinary enhancement of TMOKE can be realized through the help of the parameter optimization provided by the GA. Second, we used changes of the order of $10^{-3}$ in the refractive index of the incident medium to monitor shifts in the TMOKE peak. Significantly, we observed that these changes follow a linear behavior, making it suitable for sensing purposes. Then, we used the absolute value of the slope (widely known as sensitivity in sensing applications) as the fitness function to search for the optimum geometrical parameters that simultaneously produce high TMOKE values with the maximum sensitivity. Our results indicate a sensitivity higher than 56 nm/RIU (refractive index unit) with a figure of merit (FoM) in the order of $10^{2}$. Therefore, it is expected that our proposal has great potential for building future highly-integrated magnetoplasmonic sensing devices.

## 2. Methodology

### 2.1. Materials

**Figure 1:**
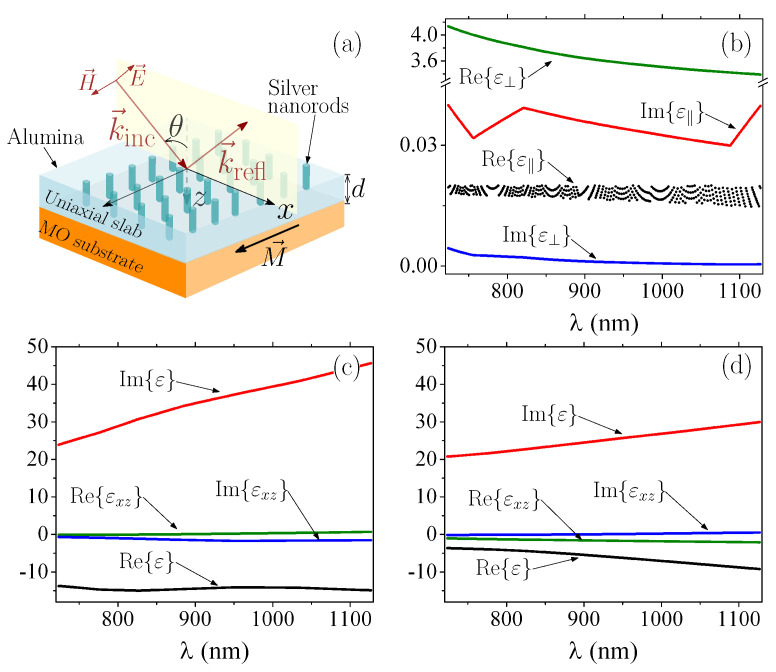
(**a**) Schematic representation of the uniaxial magnetophotonic metasurface. The *p*-polarized incident light is also illustrated, with M placed transverse to the incident plane. (**b**) ε-near-zero permittivity components of the uniaxial slab, of thickness *d*, as function of the free-space incident wavelength (λ). (**c**) and (**d**) show the permittivity components for the MO substrate when made of Co and Fe, respectively.

ε-near-zero behavior can be achieved using transparent conducting oxides [12,29] (e.g., indium tin oxide and Al-doped zinc oxide) or uniaxial metamaterials [24,30] that work near the plasma frequency, i.e., the frequency where the real part of the permittivity changes its sign. Although the option of using conductive oxide materials seems simpler at first glance, their electromagnetic responses are highly dependent on manufacturing temperature and doping, which imposes several conditions on the manufacturing process. On the contrary, the electromagnetic responses of single layers comprising plasmonic nanorods, immersed in a dielectric host, only depend on the geometry of the system, which can be easily tuned with available experimental techniques [30]. Therefore, we consider an uniaxial slab (of thickness *d*) composed by Ag nanorods in alumina (Al${}_{2}$O${}_{3}$), as schematically represented in Figure 1a. The permittivity tensor for these uniaxial materials can be written as [24]

(2) $$\hat{\varepsilon }=\left(\begin{array}{ccc} \varepsilon_{⊥} & 0 & 0\\ 0 & \varepsilon_{⊥} & 0\\ 0 & 0 & \varepsilon_{\|} \end{array}\right),$$

where the components parallel and perpendicular to the anisotropy axis (*z*-axis here) are indicated by the subscripts ‖ and ⊥, respectively, with

(3) $$\begin{array}{ccc} \varepsilon_{⊥} & = & \frac{\varepsilon_{h}\left(\varepsilon_{h}+\varepsilon_{r}\right)\left(1-f\right)+2f\varepsilon_{r}\varepsilon_{h}}{2f\varepsilon_{h}+\left(\varepsilon_{r}+\varepsilon_{h}\right)\left(1-f\right)}, \end{array}$$

(4) $$\begin{array}{ccc} \varepsilon_{\|} & = & \varepsilon_{h}\left(1-f\right)+f\varepsilon_{r}. \end{array}$$

Here, *f* represents the volume fraction of metallic inclusions per unit cell, commonly called the filling factor. $\varepsilon_{r}$ and $\varepsilon_{h}$ represent the permittivities of the Ag nanowires and Al${}_{2}$O${}_{3}$, respectively, used in agreement with the experimental results in [31].

Conventional prism-coupler-based SPR excitation uses a high-refractive-index (HRI) incident medium, commonly called prism, placed above a metallic thin film that is surrounded by the analyte medium on the opposite side. In this approach, there is an angle (called the critical angle) above which the surface wave excited by attenuated total internal reflection (widely known attenuated total reflection (ATR)) phenomenon matches the phase of the surface plasmon wave in the metallic surface. It is just under this last phase-matching condition that SPR excitation occurs in the prism-based mechanism. In contrast, we are interested in the use of an ε-near-zero slab placed above a metallic surface. Since the refractive index of air (n=1) is higher than the one for the ε-near-zero slab, SPRs can be excited even by light directly impinging from air [12]. Although similar to the attenuated total internal reflection, this last effect is know as total external reflection analogous to X-ray optics [32]. In the schematic representation of Figure 1a, reflection occurs along the *z*-axis; therefore, we performed a numerical sweep of the values of *f* as a function of the free-space incident wavelength (λ) that satisfy the condition $0<\varepsilon_{\|}⋘1$. The results of this sweep associated with Reε‖-near-zero were represented by a set of points rather than a smooth line, as pointed out in Figure 1b. The corresponding values for Imε‖, Reε⊥ and Imε⊥ are also shown in Figure 1b. The substrate was magnetized perpendicular to the plane of incidence, as illustrated in Figure 1a, with its permittivity written as

(5) $${\hat{\varepsilon }}_{\mathrm{MO}}=\left(\begin{array}{ccc} \varepsilon & 0 & im\varepsilon_{xz}\\ 0 & \varepsilon & 0\\ -im\varepsilon_{xz} & 0 & \varepsilon \end{array}\right),$$

where m=±1 indicates that M points along the ±y-axis. Calculations in this work are shown for Fe and Co, with their values (shown in Figure 1c,d) used from the experimental data in Refs. [33,34].

### 2.2. GA for TMOKE and Sensitivity Optimization

An SMM-based algorithm can be used to calculate the reflectances associated with ±M and, using the Equation (1), the corresponding TMOKE values [24]. However, this calculation mechanism becomes challenging when we are interested in optimizing the ε-near-zero structure for magnetometry (with optimized TMOKE) or sensing (with optimized sensitivity). In the first case, we should solve the analytical Equation [24] of $R_{p}\left(\pm M\right)$ for each possible combination of *d* and θ (within the sets 0<Reε‖⋘1 and 0<Imε‖⋘1 in Figure 1) searching for $\left|\mathrm{TMOKE}\right|\approx 1$. In the second (and more complex) scenario, we need to find the maximum response of the TMOKE peaks to small changes in the refractive index of the incident medium. In order to perform this, the number of points (λ,d,θ) where the equation of $R_{p}\left(\pm M\right)$ must be solved scales by at least one order of magnitude, which ends up hindering research on these structures.

**Figure 2:**
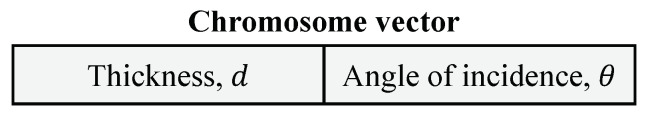
The chromosomes are represented by two-element vectors, where the first element (or gene) denotes the thickness *d* of the uniaxial slab and the second one, the angle of light incidence θ.

**Figure 3:**
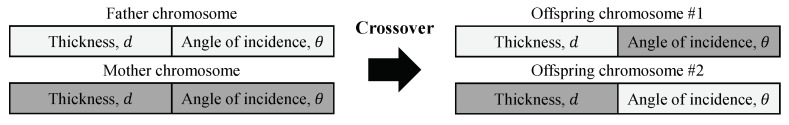
As the figure shows, the crossover operation, also known as recombination, is used to combine the genetic information of two chromosomes, called parents, into two new offspring. The recombination is realized randomly choosing two chromosomes out of the set with the fittest chromosomes, $N_{\mathrm{fitness}}$. Different colors are used to illustrate the recombination procedure.

**Figure 4:**
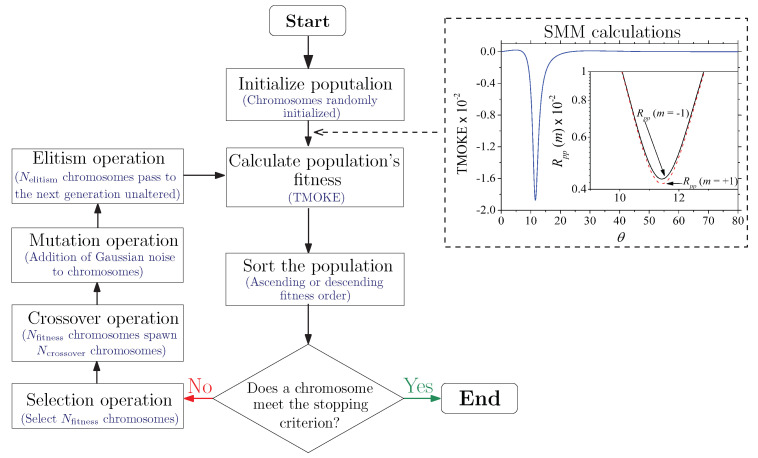
The flow chart of the GA used for the design of ε-near-zero magnetophotonic nanostructures with optimized TMOKE values. The dashed rectangle at the top right side shows the calculation of TMOKE and reflectances $R_{p}\left(m=\pm 1\right)$ (in the inset), using the SMM algorithm, for a randomly generated population of chromosomes in the first step on the left side. This last procedure is illustrated using an initially selected arbitrary chromosome.

To overcome these limitations, we used a GA that optimized the ε-near-zero magnetophotonic nanostructure. Since the substrate and the incident medium are semi-infinite, the geometry of the structure is defined by the thickness *d* of the uniaxial slab (see Figure 1). Therefore, the GA began by randomly creating an initial population of “chromosomes” (that is, comprising a set of points (d,θ) ), as depicted in Figure 2, and then performed selection, crossover (see Figure 3 for further details), mutation, and elitism operations to evolve based on the fitness function until at least one of the chromosomes met the predefined stopping criterion, as illustrated in Figure 4. This last figure specifically shows the flow chart (in the left panel) of the GA used to design a magnetophotonic nanostructure exhibiting optimized TMOKE, i.e., the TMOKE equation was employed as the guide (the fitness function) to search for the optimal parameters’ values. The GA was developed in Matlab, and the numerical results of TMOKE (the fitness function) were automated by linking the GA with an SMM-based algorithm (also in Matlab), as shown in the right panel of Figure 4. At first, the GA determined parameters such as population size (*N*), the number of chromosomes that survive and pass to the next generation ($N_{\mathrm{fitness}}$), the number of chromosomes to be created from the crossover ($N_{\mathrm{crossover}}$), the mutation rate ($p_{m}$), the standard deviation of the mutation random variable ($\sigma_{mt}$), and the number of fittest chromosomes from the current generation that are passed (unaltered) to the next one ($N_{\mathrm{elitism}}$) due to the elitism operation. The parameter $\sigma_{mt}$ is the standard deviation of the zero-centered Gaussian random variables added to the chromosome elements (e.g., *d* and θ) to mutate them. Subindex mt refers to the mutation operation. The initial chromosome population $\left\{\left[d_{1},\theta_{1}\right],\left[d_{2},\theta_{2}\right],\left[d_{3},\theta_{3}\right],\ldots ,\left[d_{j},\theta_{j}\right]\right\}$ was created from uniformly distributed random variables, with their values drawn, respectively, from the closed intervals $d=\left[5\;\;\mathrm{nm},810\;\;\mathrm{nm}\right]$ and $\theta =\left[0^{\circ },80^{\circ }\right]$, whilst the other parameters were defined as $N=10^{4}$, $N_{\mathrm{fitness}}=10^{2}$, $N_{\mathrm{crossover}}=9900$, $p_{m}=0.5$, $\sigma_{mt}=0.1$, and $N_{\mathrm{elitism}}=N_{\mathrm{fitness}}=100$ chromosomes.

The flow chart for optimizing ε-near-zero magnetophotonic nanostructures for sensing applications was mostly the same as the one depicted in Figure 4. However, the chromosomes in this case were vectors with the following elements $\left(d,\lambda ,\theta_{1},\theta_{2}\right)$, where $\theta_{1}$ and $\theta_{2}$ were used for the angles associated to the TMOKE peaks for refractive indices (of the incident medium) $n_{1}=1.000$ and $n_{2}=1.009$, respectively. The fitness function was also changed to $\left|{\mathrm{TMOKE}}_{1}\right|\times \left|{\mathrm{TMOKE}}_{2}\right|\times \left|S\right|$, where $\left|{\mathrm{TMOKE}}_{1}\right|$ and $\left|{\mathrm{TMOKE}}_{2}\right|$ represented the amplitudes at $\theta_{1}$ and $\theta_{2}$ (i.e., there were 3D functions of $\left(d,\lambda ,\theta_{1}\right)$ and $\left(d,\lambda ,\theta_{2}\right)$, respectively), and $\left|S\right|=\left|\frac{\theta_{2}-\theta_{1}}{n_{2}-n_{1}}\right|=\left|\frac{\Delta \theta }{\Delta n}\right|$ was the sensitivity. Nevertheless, to obtain the maximum values of $\left|S\right|$ we had to relax the condition $\left|\mathrm{TMOKE}\right|\approx 1$ to $\left|{\mathrm{TMOKE}}_{1}\right|\geq 0.5$ and $\left|{\mathrm{TMOKE}}_{2}\right|\geq 0.45$, which were still considered high enough to provide sharp peaks. For outstanding sensitivity values (when compared with the previous literature [23,24]), we used $\left|S\right|\geq 50$ as a stop criterion (in the fitness function).

## 3. Results and Discussion

We discuss the optimization of the ε-near-zero magnetophotonic nanostructure for giant TMOKE enhancement. The flow chart of the corresponding GA is shown in the left panel of Figure 4. For each λ, the GA randomly generated $10^{4}$ points (d,θ) and passed them to an SMM algorithm to calculate the reflectances and TMOKE, as illustrated in the right panel of Figure 4. Then, the GA selected the $N_{\mathrm{fitness}}=10^{2}$ individuals with $\left|\mathrm{TMOKE}\right|>0.96$, which were used in pairs to create $N_{\mathrm{crossover}}=9900$ new chromosomes through mutation operations between their genes. The optimization process was performed along the entire 700nm≤λ≤1150nm incident wavelength range.

**Figure 5:**
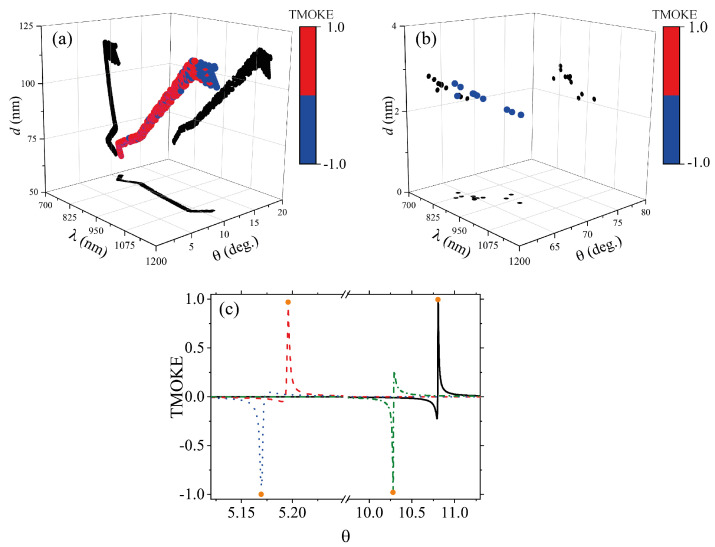
Multidimensional plot of GA optimized ε-near-zero magnetophotonic nanostructures for giant TMOKE amplitudes. Each point in the 3D plot represents a set of values (λ,d,θ,TMOKE), where the color scale is used to represent the fourth dimension on the graph (corresponding to the TMOKE value). For visualization purposes, the results are shown for (**a**) $0^{\circ }\leq \theta \leq 20^{\circ }$ and (**b**) $60^{\circ }\leq \theta \leq 80^{\circ }$. For eye-guide, we plotted projections along the (d,θ), (λ,θ) and (λ,d) planes with solid black dots. A comparison of the GA results (λ,d,θ) with the exact numerical results from the SMM is shown in (**c**) for the set of values (818.83 nm, 71.998 nm, 10.807 degree), (830.72 nm, 307.49 nm, 5.196 degree), (846.58 nm, 315.5 nm, 5.1696 degree), and (873.33 nm, 81.563 nm, 10.279 degree), whereas the solid, dashed, dotted, and dash-dotted lines, represent the exact SMM calculations for the corresponding structures. All the results in this figure were obtained for the system with the Co-substrate. Analogous results (not shown here) were obtained for the system with the Fe-substrate.

Figure 5a,b show the best fitness after an average of 15 iterations, which were carried out using a 2.5 GHz dual-core CPU with 8 GB of RAM memory in an average time of 2 min, while each iteration took approximately 8 s to be completed. Importantly, these results of the GA optimization showed that, using ε-near-zero magnetophotonic nanostructures, the maximum $\left|\mathrm{TMOKE}\right|$ values were limited to two specific ranges of θ, namely $0^{\circ }\leq \theta \leq 20^{\circ }$ and $60^{\circ }\leq \theta \leq 70^{\circ }$, as can be seen from Figure 5a,b, respectively. Moreover, we can observe that resonant angles θ≥60 only occurred for d≤5nm, whilst $\theta \leq 20^{\circ }$ were for d≥50nm. This new information, obtained through the GA optimization mechanism, is of crucial importance to guide the design and development of future highly integrated nanophotonic devices for magnetometry. In order to confirm, in Figure 5c, we comparatively plotted the results from the GA with the exact results from the SMM algorithm. Solid circles were used to denote a set of GA optimized results (λ,d,θ), which we randomly selected as (818.83 nm, 71.998 nm, 10.807 degree), (830.72 nm, 307.49 nm, 5.196 degree), (846.58 nm, 315.5 nm, 5.1696 degree), and (873.33 nm, 81.563 nm, 10.279 degree), whereas the solid, dashed, dotted, and dash-dotted lines, respectively, represented the exact SMM calculations for the corresponding structures.

**Figure 6:**
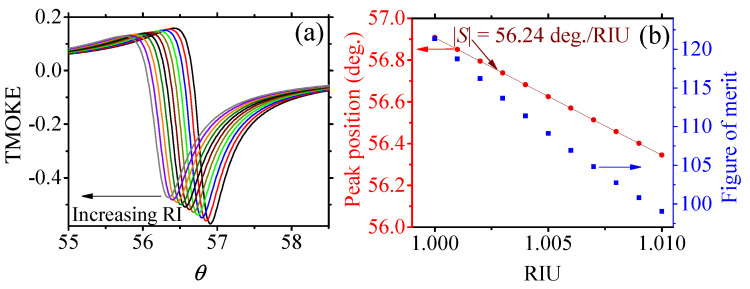
(**a**) TMOKE as a function of θ for an increasing refractive index of the incident medium. The arrow is used to indicate the successive peaks from the highest to the lowest one (associated with the refractive indexes in (**b**)). (**b**) The left vertical axis corresponds to the angular peak positions and their linear fitting, with $\left|S\right|=56.24^{\circ }/\mathrm{RIU}$, whereas the right vertical axis shows the corresponding FoM associated with each peak.

Using the previously developed GA, we optimized the structure with an Fe substrate for sensing applications. In this case, we wanted to monitor small shifts in the resonant angle θ due to small changes in the surrounding dielectric environment. Since these changes affect the quality of the resonance, the TMOKE amplitudes tend to exhibit a rapid decreasing behavior [23,24]. Furthermore, the decrease in amplitude is also detrimental to the quality of the resonance due to the broadening of the resonance peak. Therefore, we identified that the condition $\left|\mathrm{TMOKE}\right|\approx 1$ must be relaxed when we are interested in sensing applications. To cover a wide range of geometries, we redefined the initial TMOKE amplitude satisfying the condition $\left|{\mathrm{TMOKE}}_{1}\right|\geq 0.5$ (which is still considered high) as a fundamental part of the fitness function. For competitive high-resolution gas sensing platforms, we used changes in the surrounding dielectric permittivity ranging from $10^{-3}$ to $9\times 10^{-3}$, which enable detection of very low concentrations of the corresponding analyte [35]. The second fitness parameter was defined as $\left|{\mathrm{TMOKE}}_{2}\right|\geq 0.45$, corresponding to the TMOKE amplitude for the maximum change ($9\times 10^{-3}$) in the surrounding dielectric environment. This latter value, which is used to take into account the drop in TMOKE amplitude due to the resonance shift, is associated with the maximum resonant angle shift (labeled $\theta_{2}$). As we were searching for ε-near-zero magnetophotonic nanostructures with outstanding sensitivity values (in comparison to the previous literature), we also used $\left|S\right|\geq 50$ as a fitness parameter. Hence, the fitness function for the GA optimization of our sensing device was defined as $\left|{\mathrm{TMOKE}}_{1}\right|\times \left|{\mathrm{TMOKE}}_{2}\right|\times \left|S\right|$ to simultaneously satisfy the three conditions. The GA then search for optimized chromosomes ($d,\lambda ,\theta_{1},\theta_{2}$) satisfying the fitness condition. The optimization using the GA was performed with the same CPU as in the previous calculations, with an average time of 5 min. The increase in computation time was expected due to the larger search space and set of data to be analyzed in this case. The GA optimized structure for sensing had the parameters ($d,\lambda ,\theta_{1},\theta_{2},\left|S\right|)=(5\;\mathrm{nm},1125.04\;\mathrm{nm},56.89^{\circ },56.34^{\circ },56.63^{\circ }/\mathrm{RIU}$). Indeed, we confirmed this result using the exact SMM calculations, as shown in Figure 6. The numerical data of TMOKE for different refractive indices of the incident medium are shown in Figure 6a. Calculations were performed using a refractive index step of 0.001 for *n* from 1.000 to 1.009. On the left vertical axis of Figure 6a, we show the peak position (resonant θ value) as a function of *n*, from where a perfect linear behavior can be seen. The corresponding sensitivity (absolute value of the slope) for this structure was found as $56.24^{\circ }/\mathrm{RIU}$ (in excellent agreement with the GA optimization results). In addition, on the right vertical axis we show the corresponding figure of merit (FoM=S/Γ, where Γ is the TMOKE line width for each *n*), which, as noticed (in the order of $10^{2}$), is competitive with the current SPR approaches [36]. It is worth mentioning that we tried to optimize the structure with a Co-substrate for sensing. However, the sensitivities for this case were not larger than ≈$10^{\circ }/\mathrm{RIU}$, with poor FoM, which was due to the higher level of losses in the diagonal and off-diagonal permittivity components (as seen from Figure 1).

## 4. Conclusions

To summarize, we developed a GA (integrated with an SMM algorithm) to design magnetoplasmonic ε-near-zero nanostructures with maximum TMOKE amplitudes and/or optimized sensitivity and resolution. The application in sensing was illustrated with refractive index changes in the order of $10^{-3}$, suitable for high-resolution gas sensing, where an FoM in the order of $10^{2}$ was obtained along the entire refractive index sensing range. The results from the GA were compared with the exact numerical calculations using the SMM. Significantly, our GA designed these magnetoplasmonic ε-near-zero nanostructures in times ranging from 2 to 5 min using a simple dual-core CPU computer, i.e., without needing complex clusters or graphical processing units (GPUs). This last result indicates the cost-effectiveness, feasibility, and usability of our approach for the broad scientific community.

## Data Availability

Not applicable.
